# Intermittent fasting, fatty acid metabolism reprogramming, and neuroimmuno microenvironment: mechanisms and application prospects

**DOI:** 10.3389/fnut.2024.1485632

**Published:** 2024-10-24

**Authors:** Anren Zhang, Junyu Wang, Yinuo Zhao, Yu He, Nianyi Sun

**Affiliations:** ^1^Department of Rehabilitation, Shanghai Fourth People’s Hospital, School of Medicine, Tongji University, Shanghai, China; ^2^Department of Rehabilitation, Shengjing Hospital of China Medical University, Shenyang, China

**Keywords:** intermittent fasting, fatty acid metabolism, neuroimmuno microenvironment, AMPK, SIRT1, ketone bodies, autophagy

## Abstract

Intermittent fasting (IF) has demonstrated extensive health benefits through the regulation of fatty acid metabolism and modulation of the neuroimmune microenvironment, primarily via the activation of key signaling pathways such as AMP-activated protein kinase (AMPK) and sirtuin 1 (SIRT1). IF not only facilitates fatty acid oxidation and improves metabolic health, but also enhances mitochondrial function, mitigates oxidative stress, promotes autophagy, and inhibits apoptosis and ferroptosis. These mechanisms contribute to its substantial preventive and therapeutic potential in various conditions, including neurodegenerative disorders such as Alzheimer’s and Parkinson’s diseases, autoimmune diseases, and neurotraumatic conditions. While supportive evidence has been obtained from animal models and preliminary clinical studies, further large-scale, long-term randomized controlled trials are imperative to establish its safety and evaluate its clinical efficacy comprehensively.

## 1 Introduction

Intermittent fasting (IF), a dietary pattern characterized by complete or partial abstinence from food during specific time periods, has gained increasing global attention and research interest in recent years. IF has shown significant positive effects on weight management and metabolic health and has demonstrated potential in the prevention and treatment of various chronic diseases (1). Studies have indicated that IF can improve metabolic health through several mechanisms, including enhanced insulin sensitivity, reduced blood pressure, and improved lipid profiles (2, 3). Furthermore, IF has exhibited notable effects in promoting cardiovascular health, preventing cancer, and enhancing resistance to neurological disorders (4, 5).

Fatty acid metabolism plays a crucial role in maintaining energy homeostasis and overall health, as the synthesis and breakdown of fatty acids provide essential energy and metabolic intermediates for the body (6). Fatty acids are not only key components of cell membranes but also participate in signal transduction, regulation of gene expression, and inflammatory responses (7). Dysregulation of fatty acid metabolism is closely associated with various metabolic diseases, such as obesity, diabetes, and cardiovascular diseases (8).

Recent research has highlighted that IF can significantly impact fatty acid metabolism by modulating processes such as fatty acid breakdown, oxidation, and synthesis, thereby contributing to metabolic reprogramming (9–13). Beyond its effects on metabolic health, intermittent fasting (IF) has also garnered considerable attention for its regulation of the neuroimmune microenvironment. The neuroimmune microenvironment refers to the complex network of interactions between the nervous and immune systems, which plays a critical role in neuroinflammation, neuroprotection, and repair processes. IF regulates neuroimmune responses by influencing metabolic products derived from fatty acid metabolism, such as ketone bodies and short-chain fatty acids (SCFAs) (14, 15). For instance, ketone bodies reduce oxidative stress and protect neurons by activating the Nrf2 pathway (16–19), whereas SCFAs modulate neuroinflammation through regulation of T-cell and microglial activity (20–22).

In addition, IF significantly impacts metabolic status and neuroprotection mechanisms by activating signaling pathways such as AMPK and SIRT1, which promote fatty acid breakdown while inhibiting synthesis (9–13). AMPK, as an energy sensor, maintains cellular energy homeostasis primarily by promoting fatty acid oxidation and reducing fatty acid synthesis (23). Meanwhile, SIRT1 enhances mitochondrial function and promotes autophagy through the regulation of multiple metabolic genes (24). These mechanisms are crucial for the potential therapeutic effects of IF in neurodegenerative diseases (e.g., Alzheimer’s and Parkinson’s disease), autoimmune diseases, and neurotraumatic conditions. Besides its influence on neuroinflammation and metabolic reprogramming, IF also plays a key role in neuroprotection by modulating mitochondrial function, reducing oxidative stress, and balancing autophagy, apoptosis, and ferroptosis (25–27). For example, IF mitigates oxidative damage to neurons by reducing reactive oxygen species (ROS) production and enhancing antioxidant systems (27), thus demonstrating potential in the prevention and treatment of neurodegenerative diseases (28, 29).

In recent years, an increasing number of animal studies and preliminary clinical trials have investigated the impact of IF on fatty acid metabolism and the neuroimmune microenvironment, revealing its promising applications in neurodegenerative, autoimmune, and neurotraumatic diseases (30–33). However, current research still faces limitations, particularly in elucidating the specific molecular mechanisms and assessing the long-term safety of IF. Therefore, this review aims to systematically summarize the mechanisms by which IF affects fatty acid metabolism and regulates the neuroimmune environment, providing a theoretical foundation and scientific guidance for its clinical application.

## 2 Intermittent fasting and fatty acid metabolism

**Figure 1:**
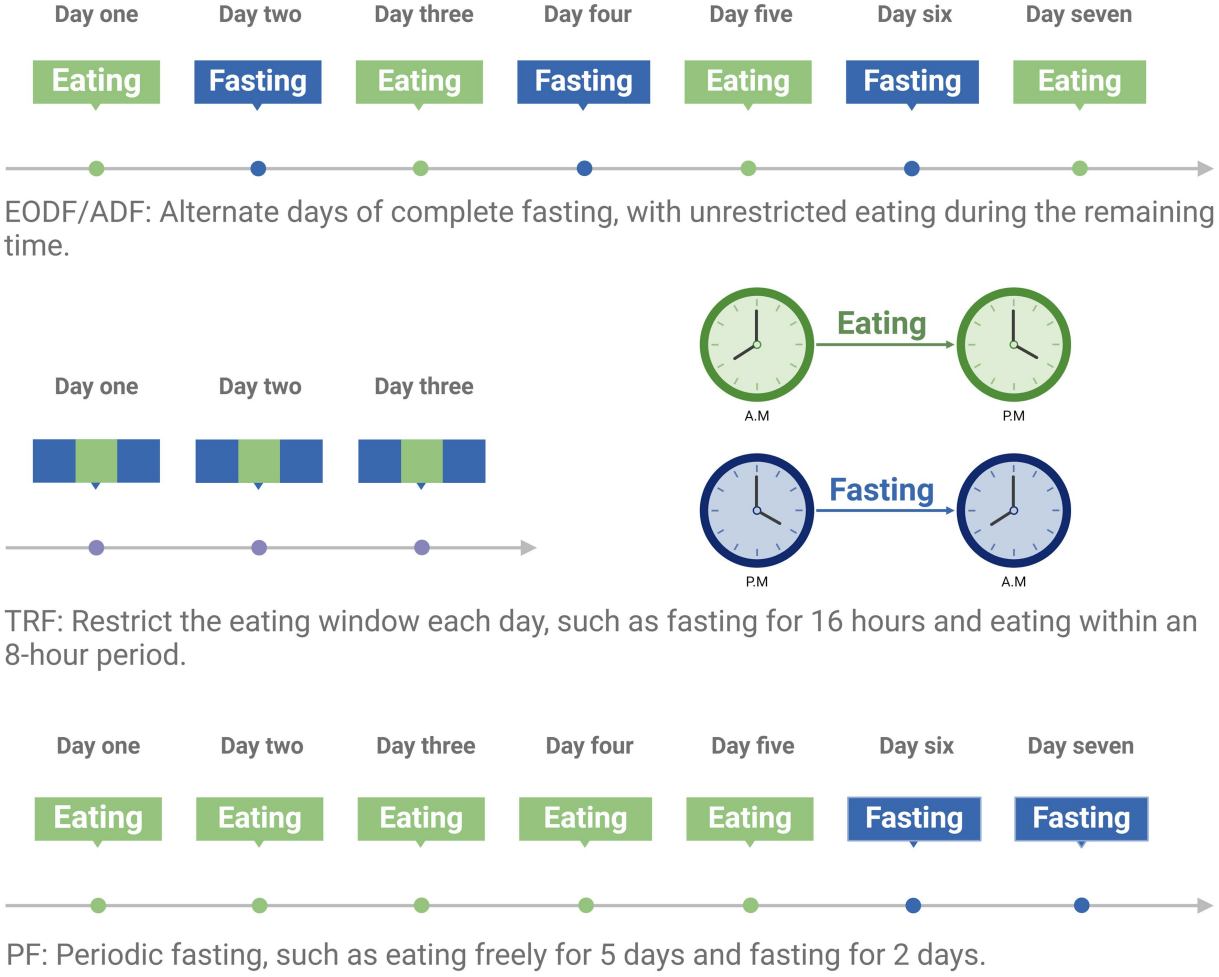
Dietary pattern of intermittent fasting. Created with BioRender.com.

Intermittent Fasting is a dietary pattern where complete or partial fasting occurs during specific periods, followed by periods of unrestricted eating (*ad libitum*, AL). Common IF patterns include Every-other-day fasting (EODF), whole-day fasting (1 or 2 days of fasting per week), time-restricted feeding (TRF, where energy intake is typically limited to an 8–12 h window), and periodic fasting (PF, fasting for 2 days following 5 days of unrestricted eating) (29) (Figure 1). IF has a wide range of physiological effects on the body, including improving glucose metabolism, promoting fat breakdown, and reducing inflammation. It also significantly affects the cardiovascular, nervous, and immune systems. For example, IF can improve cardiovascular health, enhance neuroprotection, and modulate immune responses to reduce inflammation (34).

Fatty acids are aliphatic organic compounds with a carboxyl end. Different classification criteria result in different types of fatty acids, typically categorized by saturation level, such as saturated fatty acids, monounsaturated fatty acids, and polyunsaturated fatty acids. Polyunsaturated fatty acids can be further classified into *ω*-3 and ω-6 fatty acids based on the position of the first double bond in the carbon chain relative to the methyl end (35–38). Fatty acids are both key components of lipids and vital elements of cell membrane structure, maintaining membrane integrity, fluidity, and function. They also serve as energy sources through the tricarboxylic acid cycle, converting into ATP to fuel daily activities. However, with the rise of metabolomics and ongoing research into disease mechanisms, it has been found that fatty acids play critical roles in many disease-related metabolic disorders, such as inflammation, diabetes, cystic fibrosis, asthma, tumors, and cancer (35–38). Fatty acid metabolism includes the synthesis and oxidation processes, primarily occurring in the liver, involving key enzymes like fatty acid synthase; fatty acid oxidation mainly occurs in mitochondria through the *β*-oxidation pathway, breaking down fatty acids into acetyl-CoA to provide energy (39).

IF regulates fatty acid metabolism through various mechanisms, including activating AMPK (AMP-activated protein kinase) and SIRT1 (Sirtuin 1) signaling pathways, which promote fatty acid breakdown and inhibit synthesis. Additionally, during fasting, the body undergoes metabolic reprogramming, generating more ketone bodies to supply energy (9–13). Recent studies indicate that the immune and metabolic systems are highly integrated, with immune cell function itself being regulated in coordination with cellular metabolism. For example, after inflammation activation, macrophages exhibit glucose and fatty acid oxidative metabolism and acquire an anti-inflammatory phenotype in the context of tissue repair and remodeling (14, 35, 40). IF significantly affects the levels of metabolic products, such as increasing *β*-hydroxybutyrate and short-chain fatty acids (SCFAs), which play crucial roles in regulating inflammation, oxidative stress, and energy metabolism (41–43).

## 3 Fatty acid metabolism and neuroimmune microenvironment

The neuroimmune microenvironment refers to the complex interaction network between the nervous and immune systems. This microenvironment plays a crucial role in neuroinflammation, neuroprotection, and repair. Metabolites from fatty acid metabolism, such as ketone bodies and SCFAs, are vital in regulating neuroimmune responses (14, 15). Fatty acid metabolism affects neuroimmune microenvironment by regulating signaling pathways like PPARs (Peroxisome Proliferator-Activated Receptors), NF-κB (Nuclear factor kappa-light-chain-enhancer of activated B cells), and Nrf2 (Nuclear factor E2-related factor 2), influencing the functions of neural and immune cells (16–18). For example, ketone bodies can reduce oxidative stress and protect neurons by activating the Nrf2 pathway; SCFAs can regulate neuroinflammation by modulating T cells and microglia (19–22). Polyunsaturated fatty acids play a vital role in regulating inflammation. The anti-inflammatory effects of *ω*-3 fatty acids are well-established (44, 45), attributed mainly to (1) their conversion into pro-resolving mediators like lipid regulators, resolvins, and anti-inflammatory mediators, which inhibit pro-inflammatory factors like TNF-a, IL-1β, and NF-κB, and (2) the inhibition of *ω*-6 fatty acids from converting into pro-inflammatory substances like prostaglandins (PG) E2 and leukotrienes (LT) B4. In contrast to the anti-inflammatory effects of ω-3 fatty acids, ω-6 fatty acids exhibit pro-inflammatory effects, being converted into PGE1 or lipoxin A4, which promote inflammation (46). Regardless of their pro- or anti-inflammatory roles, fatty acids are crucial in the initiation, progression, and resolution of inflammation (47).

The interactions between the neuroimmune system and neurons play a vital role in maintaining homeostasis within the central nervous system. IF, by modulating fatty acid metabolism, significantly influences the neuroimmune environment, thereby affecting neuronal health. The study by Pak et al. (48) demonstrated that IF can protect mitochondrial function in neurons, reverse neuronal damage, and potentially benefit the treatment of future neurological disorders. Additionally, IF enhances the ability of neurons to resist ferroptosis by upregulating antioxidant factors such as glutathione peroxidase 4 (Gpx4) (49). Therefore, changes in fatty acid metabolism indirectly promote neuronal survival and functional recovery by regulating the neuroimmune environment, underscoring its importance for neuroprotection.

## 4 Interactions between IF, fatty acid metabolism, and neuroimmune regulation

**Figure 2:**
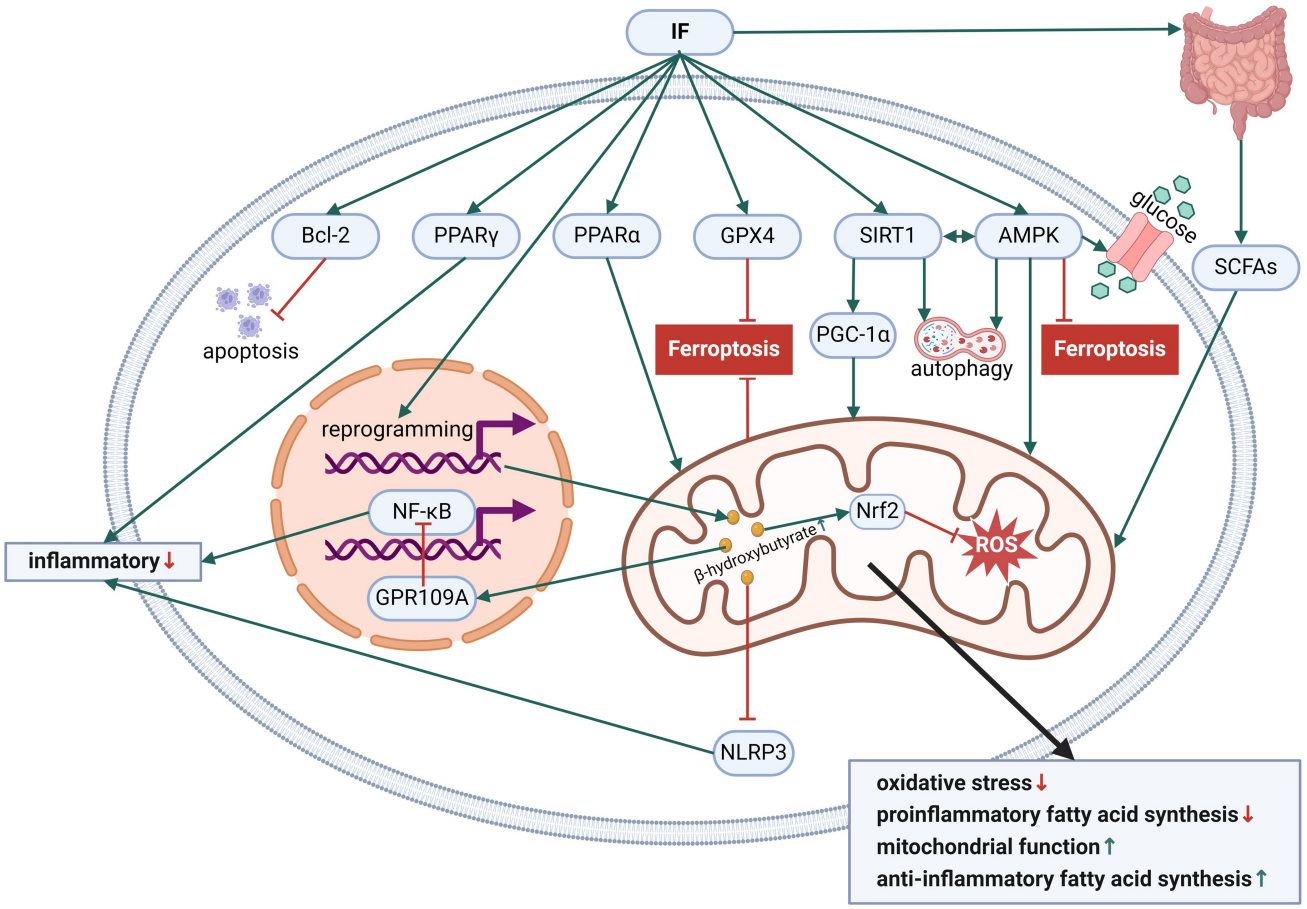
The effects of IF on fatty acid metabolism and neuroimmune regulation. Created with BioRender.com.

Figure 2 has presented the effects of IF on fatty acid metabolism and neuroimmune regulation.

### 4.1 Activation of AMPK and SIRT1 signaling pathways

IF influences fatty acid metabolism through various mechanisms, thereby regulating the neuroimmune microenvironment. First, IF can activate AMPK and SIRT1 signaling pathways, promoting fatty acid breakdown and inhibiting fatty acid synthesis (9–13). AMPK, as the primary regulator of cellular energy balance, improves metabolic health by promoting fatty acid oxidation and reducing fatty acid synthesis (23). SIRT1, through deacetylation, regulates the expression of various metabolic genes, enhancing fatty acid oxidation and mitochondrial function (24). Studies show that AMPK acts as a sensor of cellular energy status, being activated when energy is insufficient, promoting fatty acid oxidation and glucose uptake, thereby increasing energy supply (23, 50). SIRT1 enhances mitochondrial biogenesis and function through deacetylating PGC-1α (Peroxisome proliferator-activated receptor gamma coactivator 1-alpha), improving cellular metabolic status (51, 52). Moreover, the synergistic effect of AMPK and SIRT1 significantly enhances fatty acid oxidative metabolism, increasing cellular energy utilization efficiency (23, 53).

### 4.2 Metabolic reprogramming and ketone body production

IF induces a metabolic reprogramming state in the body, significantly increasing the production of ketone bodies (such as *β*-hydroxybutyrate). Ketone bodies are products generated in the liver during fatty acid breakdown and can serve as alternative energy sources for the brain and other tissues (19, 54). As an important ketone body, β-hydroxybutyrate has antioxidant and anti-inflammatory effects, reducing oxidative stress and protecting neurons by activating the Nrf2 signaling pathway (19, 55). Additionally, ketone bodies can reduce neuroinflammation by inhibiting the NLRP3 inflammasome (56–58). Research has shown that the role of ketone bodies in energy metabolism extends beyond being an alternative energy source, as they also regulate cellular function through various signaling pathways. For example, *β*-hydroxybutyrate inhibits the NF-κB signaling pathway by activating GPR109A (G protein-coupled receptor 109A), reducing inflammation (59, 60). Moreover, ketone bodies can also inhibit HDAC (Histone deacetylase), increasing the expression of antioxidant enzymes and further reducing oxidative stress (61–63).

### 4.3 SCFAs and gut microbiota

IF influences the composition and function of gut microbiota, regulating the production of SCFAs, which have significant effects on the neuroimmune microenvironment (13, 42, 43). SCFAs, such as acetate, propionate, and butyrate, are metabolites produced by gut microbiota fermenting dietary fiber and play key roles in regulating immune responses, maintaining gut barrier function, and promoting energy metabolism (64). For example, butyrate can regulate immune cell function and reduce neuroinflammation by activating GPR41 and GPR43 receptors (6, 65). The diversity and health status of the gut microbiota play crucial roles in regulating host immune responses. IF significantly alters the diversity and abundance of gut microbiota, increasing beneficial bacteria like Bifidobacterium and Lactobacillus while reducing harmful bacteria like Bacteroides (66–68). These changes not only improve gut health but also enhance resistance to neuroinflammation by regulating the host immune system (69).

### 4.4 PPARs and NF-κB signaling pathways

PPARs are ligand-activated transcription factors belonging to the nuclear hormone receptor superfamily and are key regulators of various pathophysiological processes related to energy metabolism, including lipid, carbohydrate metabolism, and inflammation. Three PPAR isoforms have been identified so far, commonly referred to as PPARα, PPARβ/*δ*, and PPARγ, which show different tissue distributions and ligand specificities (70, 71). Among them, PPARγ plays a broad regulatory role in inflammation, particularly in inhibiting inflammatory responses through competitive inhibition of inflammatory signaling pathways, regulating the generation of inflammatory and anti-inflammatory mediators, reducing reactive oxygen species release, and affecting cell proliferation, differentiation, and apoptosis, which is crucial for M2 macrophage/microglial polarization (72). PPARα plays an important role in fatty acid oxidation, and IF promotes fatty acid *β*-oxidation and reduces fat accumulation by activating PPARα (73). PPARγ, on the other hand, regulates adipocyte differentiation and inflammatory responses, and IF reduces the generation of inflammatory factors by modulating PPARγ activity (74, 75). Additionally, NF-κB is a major regulator of inflammatory responses, and IF reduces neuroinflammation by inhibiting the NF-κB signaling pathway and reducing the expression of inflammatory factors (76, 77). For example, ketone bodies and SCFAs also reduce neuroinflammation by inhibiting NF-κB (78–80).

### 4.5 Mitochondrial function and oxidative stress

Studies have found that IF enhances mitochondrial function, reduces oxidative stress, and protects neurons (26). IF enhances mitochondrial biogenesis and function by activating AMPK and SIRT1, increasing ATP production and improving cellular energy supply (23, 24, 81). Additionally, IF reduces the production of free radicals and protects neurons from oxidative damage by increasing the expression of mitochondrial antioxidant enzymes (25, 82). IF also maintains mitochondrial health by regulating mitochondrial autophagy and removing damaged mitochondria (83–85). Moreover, IF increases the expression of mitochondrial fusion-related proteins (such as mitofusin-2), promoting mitochondrial fusion and enhancing mitochondrial function (86, 87). Simultaneously, IF regulates the activity of mitochondrial fission-related proteins (such as dynamin-related protein 1), maintaining mitochondrial fission and renewal (88). IF reduces oxidative stress and neuronal damage by decreasing reactive oxygen species (ROS) production and enhancing the antioxidant system (27). Excessive ROS production is a major pathological mechanism of neurodegenerative diseases, and IF protects neurons by activating the Nrf2 signaling pathway, increasing the expression of antioxidant enzymes such as superoxide dismutase (SOD) and glutathione peroxidase (GSH-Px), and reducing ROS accumulation (27). Additionally, IF further reduces ROS production by regulating the autophagy pathway and removing damaged mitochondria (28, 89).

### 4.6 Autophagy and apoptosis

Autophagy and apoptosis are important regulatory mechanisms for cellular metabolism and survival, and IF plays a key role in these processes, producing neuroprotective effects (33, 89–92). Autophagy is a mechanism that clears damaged proteins and organelles within cells and is crucial for maintaining cellular homeostasis and responding to metabolic stress (93). IF promotes autophagy by activating AMPK and SIRT1, helping to clear harmful substances in neurons and reducing neurodegenerative changes (25, 94). Apoptosis is a process of programmed cell death, and excessive apoptosis can lead to the loss of neurons (95). IF reduces neuronal death and protects the nervous system by regulating the expression of the Bcl-2 family of proteins and inhibiting apoptosis pathways (33). IF maintains cellular homeostasis and function by regulating autophagy and apoptosis pathways, which is significant for the prevention and treatment of neurodegenerative diseases (29, 89).

### 4.7 Ferroptosis

Ferroptosis is induced by the accumulation of iron-dependent lipid hydroperoxides and is closely linked to fatty acid metabolism levels (96). The study by Yang et al. (49) demonstrated that IF can alleviate oxidative stress related to fatty acid metabolism and mitochondrial dysfunction, while also inhibiting cellular ferroptosis by increasing the expression of the protective enzyme glutathione peroxidase 4 (Gpx4). Furthermore, IF promotes the activation of the AMPK pathway; Lee et al. (97) found that AMPK-mediated energy stress effectively suppresses ferroptosis. There is growing evidence that various neurodegenerative diseases, including Parkinson’s disease, stroke, Huntington’s disease, and traumatic brain injury, are associated with ferroptosis (98, 99). By inhibiting cellular ferroptosis, IF may help reduce the risk of developing these conditions.

## 5 Experimental and clinical studies

In recent years, an increasing number of animal experiments and preliminary clinical explorations have investigated the effects of IF on fatty acid metabolism and the neuroimmune microenvironment.

### 5.1 Animal experimental studies

#### 5.1.1 Neurodegenerative diseases

##### 5.1.1.1 Alzheimer’s disease

In the study by Pedersen et al. (30), APP gene-mutant mice subjected to EODF intervention died within 2–3 weeks. These mice experienced severe hypoglycemia on fasting days, leading to death. Further analysis showed significant abnormalities in the hypothalamic–pituitary–adrenal axis regulation of the stress response in APP gene-mutant mice. This abnormality was associated with the accumulation of Aβ in the cerebral cortex, hippocampus, and hypothalamus, making them unable to respond normally to various stressors, including restraint stress and surgery. Zhu et al. (100) subjected PS1 gene-knockin mice to continuous EODF for 3 months and found that the EODF group exhibited enhanced resistance to excitotoxic damage in hippocampal CA1 and CA3 neurons, with fewer lipid peroxidation products (4-hydroxy-2-nonenal) compared to the AL group, suggesting that inhibition of oxidative stress might be a potential mechanism for the neuroprotective effects of EODF. Guo et al. (101) found that adult 3-month-old SD male rats subjected to 3 months of EODF, compared to the normal diet group, retained glucose and glutamate uptake and mitochondrial function after synaptosomes were exposed to Aβ protein damage, with increased stress protein levels, suggesting that EODF may alter synaptic homeostasis and enhance resistance to damage. Contestabile et al. (102) found that 2-month-old male Wistar rats subjected to 6 months of EODF exhibited reduced acetylcholine transferase (ChAT) reduction in the frontal–parietal cortex after basal forebrain injection of amygdalin compared to the AL group, suggesting that EODF may exert partial neuroprotective effects against this damage. Halagappa et al. (103) conducted a study on 3-month-old triple-transgenic AD (3xTgAD) mice and found that EODF may exert neuroprotective effects by resisting the adverse effects of Aβ protein and tau protein on synaptic function. Zhang et al. (104) showed that IF improved cognitive dysfunction, prevented Aβ deposition in the brain, and restored AQP4 polarity in the AD mouse model (APP/PS1 double-transgenic mice). Additionally, IF downregulated the expression of AQP4-M1 and histone deacetylase 3, reduced the AQP4-M1/M23 ratio, and increased miR-130a expression in the cortical region of APP/PS1 mice. Shin et al. (105) suggested that IF could prevent the worsening of cognitive function, energy metabolism, and dyslipidemia induced by estrogen deficiency in Alzheimer’s rats. Liu et al. (106) found that in the AppNL-GF mouse model of AD, IF reduced neuronal network hyperexcitability and improved hippocampal synaptic plasticity defects in a SIRT3-dependent manner. Li et al. (107) subjected 6-month-old male 3xTg-AD and wild-type mice to IF or AL for 3 months. By bilaterally injecting lentiviral vectors carrying siRNA targeting GSK-3β into the hippocampal dentate gyrus region to modulate GSK-3β activity, the study found that IF promoted neuronal differentiation and maturation in the dentate gyrus and improved the known dysfunction in 3xTg-AD mice. These effects could be reversed by siRNA targeting GSK-3β. After IF, the insulin and protein kinase A signaling pathways were inhibited, while the adenosine monophosphate-activated protein kinase and BNDF pathways were activated. Rangan et al. (108) found that long-term fasting-mimicking diet (FMD) cycles reduced hippocampal Aβ load and tau hyperphosphorylation in E4FAD and 3xTg AD mouse models, enhanced neurogenesis, reduced the number of microglia, and decreased the expression of neuroinflammatory genes, including the superoxide-producing NADPH oxidase (Nox2). However, Lazic et al. (109) evaluated the effects of a preventive EODF regimen on the neurodegenerative phenotype of 5XFAD transgenic mice and found a significant increase in inflammation in the cortex of 5XFAD-EODF mice, with increased glial cell reactivity and/or proliferation, along with increased pro-inflammatory cytokines TNF-*α*, p38 MAPK, and EAAT2, and decreased GAD67, associated with glutamate excitotoxicity, and increased NMDA receptor subunit 2B in the cortex of 5XFAD-EODF mice. This study suggests that EODF regimens may exacerbate Alzheimer’s-like neurodegenerative and neuroinflammatory changes, indicating the need for caution when using dietary restriction during the prodromal stage of such neurodegenerative diseases.

##### 5.1.1.2 Huntington’s disease

HD is a movement control disorder caused by selective degeneration of striatal neurons. The mitochondrial toxin 3-nitropropionic acid (3NP) is commonly used to create HD animal models. Bruce-Keller et al. (110) found that after several months of EODF, adult SD male rats showed enhanced resistance to 3NP-induced striatal neuronal damage and improved motor function. Guo et al. (101) found that adult 3-month-old SD male rats subjected to 3 months of EODF, compared to the normal diet group, retained glucose and glutamate uptake and mitochondrial function after synaptosomes were exposed to 3NP, with increased stress protein levels, suggesting that EODF may alter synaptic homeostasis and enhance resistance to damage. Duan et al. (111) studied Huntington’s gene-mutant mice and found that EODF slowed disease progression, increased survival rates, improved motor performance, reduced brain atrophy, and decreased huntingtin aggregate formation and caspase activation, normalized glucose regulation, and restored BDNF and chaperone protein levels in the cortex and striatum compared to the AL group. Ehrnhoefer et al. (112) showed that a scheduled feeding paradigm was sufficient to reduce mutant huntingtin levels in YAC128 mice expressing cleavable mutant huntingtin. Wang et al. (113) divided 6-month-old Q175 mice into two groups: AL and TRF. Q175 mice subjected to TRF treatment were fed on a 6 h feeding/18 h fasting schedule. After 3 months of treatment (when the mice reached the early stage of the disease), TRF-treated Q175 mice showed improved motor activity rhythms and sleep–wake cycles. Finally, using NanoString gene expression assays, the expression of several HD-related markers in the striatum of treated mice returned to WT levels. Whittaker et al. (114) also found that the spontaneous activity and sleep behavior rhythms of TRF-treated BACHD mice improved.

##### 5.1.1.3 Parkinson’s disease

PD is a movement disorder caused by the degeneration of dopaminergic neurons in the substantia nigra. Various models are used to study PD, including *α*-synuclein-mutant mice, the toxin MPTP, striatal injection of the catecholamine neurotoxin 6-hydroxydopamine (6-OHDA), and others (115). Duan et al. (116) subjected 4-month-old male C57Bl/6 mice to either AL or EODF for 3 months, followed by MPTP administration. The results showed that EODF reduced MPTP-induced motor dysfunction and dopaminergic neuron damage in the substantia nigra, increased levels of the stress proteins HSP-70 and GRP-78 in dopaminergic neurons, and exerted neuroprotective effects. In another study, Holmer et al. (117) found that even when EODF was initiated after MPTP administration, beneficial effects still appeared, including decreased extracellular striatal glutamate levels. However, in another study using a 6-OHDA-induced PD rat model, EODF failed to prevent substantia nigra-striatal degeneration (118). Zhou et al. (119) reported that FMD accelerated motor function preservation and reduced dopaminergic neuron loss in the substantia nigra of MPTP-induced PD mice. FMD reduced the number of glial cells and the release of TNF-*α* and IL-1β in PD mice. Additionally, the study found that FMD treatment altered the composition of gut microbiota in PD mice, including increased abundance of Firmicutes, Bacteroidetes, and Verrucomicrobia, and decreased abundance of Proteobacteria at the phylum level. FMD also modulated changes in short-chain fatty acids (SCFAs) such as propionate and isobutyrate reduction, as well as butyrate and pentanoic acid and other metabolite increases induced by MPTP. Ojha et al. (120) observed that EODF attenuated MPTP-induced loss of dopaminergic neurons and astrocyte activation in the substantia nigra and striatum. Furthermore, EODF reduced MPTP-induced striatal dopamine depletion, increased phosphorylation of PI3K and Akt, and increased phosphorylation of ERK and CREB, further supporting the involvement of neurotrophic factors in the observed neuroprotection.

##### 5.1.1.4 Amyotrophic lateral sclerosis

ALS is a fatal disease characterized by progressive paralysis caused by degeneration of spinal motor neurons. Some ALS cases are caused by mutations in the Cu/Zn superoxide dismutase (SOD) gene. In contrast to the beneficial effects observed in animal models of AD, PD, and HD, Pedersen et al. (121) found that EODF did not show beneficial effects on disease onset or progression in ALS mice with the Cu/Zn SOD gene mutation, and once symptoms appeared, EODF actually accelerated disease progression. This suggests that dietary restriction may not effectively counteract all neurodegenerative diseases involving genetic changes. One possibility is that dietary restriction may produce different effects on gene expression in motor neurons compared to brain neurons. Another possibility is that dietary restriction may indeed induce the expression of neuroprotective proteins in motor neurons, such as growth factors and stress proteins, but the pathogenic pathways involved in Cu/Zn SOD mutations are not affected by dietary restriction. Further studies on the effects of dietary restriction in transgenic ALS mice may help identify the specific molecular conditions under which DR combats neurodegeneration. Charcot–Marie-Tooth type 1A (CMT1A) is the most common hereditary neuromuscular disease involving abnormal expression of peripheral myelin protein 22 (PMP22), a progressive demyelinating disease of the peripheral nervous system. Madorsky et al. (122) subjected 2-month-old Trembler J (TrJ) neuropathic mice to either AL or EODF for 5 months. The EODF group showed significant improvement in motor performance compared to the AL group, with functional benefits associated with increased myelin protein expression, thickened myelin, reduced redundant basement membrane, and reduced abnormal Schwann cell proliferation. The EODF group also showed improved nerve morphology, accompanied by reduced PMP22 aggregation and increased expression of cytosolic chaperones and components of the autophagy/lysosomal pathway.

#### 5.1.2 Autoimmune diseases

##### 5.1.2.1 Multiple sclerosis and experimental autoimmune encephalomyelitis

Razeghi Jahromi et al. (31) induced EAE in C57BL/6 mice using Myelin Oligodendrocyte Glycopeptide (MOG) 35–55, and mice were subjected to EODF after the first clinical symptoms appeared or 30 days after disease induction, for a total of 10 days. The study found that early fasting could reduce EAE severity by improving spinal cord demyelination, inhibiting IFN-*γ* and TNF-*α* secretion, and increasing IL-10 production in spleen cells. Choi et al. (123) found that periodic 3-day FMD could effectively improve demyelination and symptoms in a mouse EAE model. These improvements were associated with increased corticosterone levels and regulatory T (Treg) cells, reduced levels of pro-inflammatory cytokines, TH1 and TH17 cells, and antigen-presenting cells (APCs). Additionally, in EAE and cuprizone MS models, FMD promoted oligodendrocyte precursor cell regeneration and axonal remyelination. Cignarella et al. (124) found that IF improved the clinical course and pathology of EAE models by altering gut microbiota. IF increased the abundance of Lactobacillaceae, Bacteroidaceae, and Prevotellaceae, and enhanced antioxidant microbial metabolic pathways. IF also altered T cells in the gut, reducing IL-17-producing T cells and increasing regulatory T cells. Fecal microbiota transplantation from IF mice improved EAE in immune-receptor mice fed a normal diet. Bai et al. (125) induced EAE in C57BL/6 mice by immunizing with Myelin Oligodendrocyte Glycoprotein 35–55 peptide. After 4 weeks of EAE symptoms, mice were subjected to 3 days of modified FMD followed by 4 days of AL, for a total of two cycles. Compared to the control group, FMD mice showed significant reductions in EAE severity, spinal cord immune cell infiltration, and CNS demyelination. FMD also reversed the accumulation of total CD4+ T cells, particularly CNS-accumulating IFN-*γ*-producing CD4+ T cells, induced by EAE. Additionally, FMD increased cell proliferation rates in the CNS and enhanced the expression of brain-derived neurotrophic factor (BDNF) and remyelination markers. Wang et al. (126) reported that catecholaminergic (CA) neurons in the ventrolateral medulla (VLM) of mice were activated during fasting and demonstrated that the activity of these CA neurons influenced T cell distribution in EAE. Ablation of VLM CA neurons largely reversed fasting-induced T cell redistribution. Activation of these neurons drove T cells to home to the bone marrow via a CXCR4/CXCL12 axis-dependent mechanism, possibly mediated by a neural circuit stimulating corticosterone secretion.

##### 5.1.2.2 Other autoimmune diseases

Chen et al. (127) measured acanthosis in IMQ-induced psoriatic mice and assessed their pathological phenotype. The study found that 2 weeks of TRF reduced psoriatic-like lesions, decreased inflammatory cytokines, and alleviated immunosenescence in mice. TRF increased the number of CD4+ Treg cells in skin lesions while reducing the number of Th2 and Th17 cells in the spleen. Additionally, TRF administration led to a reduction in the number of CD4+ senescent T cells in the dermis and spleen, consistent with the reduced expression of senescence-associated genes in splenic CD4+ T cells. Chen et al. (128) found that IF significantly improved IMQ-induced psoriatic-like dermatitis and reduced the number of γδT17 cells and IL-17 production in draining lymph nodes and psoriatic lesions by inhibiting γδT17 cell proliferation and increasing apoptosis. Additionally, IF significantly reduced the number of monocytes in the blood, which was associated with reduced monocytes, macrophages, and dendritic cells in psoriatic skin inflammation. However, in the study by Hong et al. (129), contrary to expectations, IF resulted in higher levels of anti-dsDNA antibodies, immune complex deposition, and glomerular damage in the kidneys compared to the control group. Proteinuria was also aggravated in the IF group. IF increased the abundance of B cells, plasmablasts, and plasma cells in the spleen and lymph nodes, as well as increased autophagy in plasma cells. Chloroquine-induced autophagy inhibition reduced anti-dsDNA antibody secretion by splenocytes *in vitro*. These findings suggest that IF exacerbates lupus nephritis in MRL/lpr mice by increasing the formation of self-antibody immune complexes.

#### 5.1.3 Neurotraumatic diseases

##### 5.1.3.1 Spinal cord injury

In recent years, researchers from the International Collaboration on Repair Discoveries at the University of British Columbia, Canada (130–132), applied EODF to treat rats with acute incomplete cervical and thoracic spinal cord injury (SCI), obtaining positive results in behavioral assessments and evaluations of neuroprotective effects through blood *β*-hydroxybutyrate levels and BDNF levels. However, another study by the team showed (133) that, in contrast to their previous observations in SD rats, EODF did not provide beneficial effects on tissue preservation or improve hindlimb motor function in SCI mice. The research team suggested that this difference might be related to different metabolic responses to intermittent fasting. Yuan et al. (33) found that rats subjected to IF showed significant improvements in behavioral performance and neuronal survival at the spinal cord injury site, with inhibition of endogenous apoptotic pathways and upregulation of autophagy. Wang et al. (134) found that after EODF intervention, differentially expressed genes (DEGs) were enriched and associated with various biological events, including immune-inflammatory responses, cell differentiation, protein modification, neurogenesis, and apoptosis. Specifically, there were significant spatiotemporal differences in DEGs associated with neuroprotection between the EODF and AL intervention groups. These DEGs were primarily concentrated on days 1, 3, and 7 after SCI. The relative abundance of certain genera was significantly associated with DEGs related to neuroprotection in the EODF-SCI group.

##### 5.1.3.2 Stroke

Yu et al. (135) subjected 1-month-old adult SD rats to either AL or EODF for 3 months, followed by middle cerebral artery occlusion (MCAO). The results showed that EODF rats had significantly reduced infarct volumes and improved neurological performance. Arumugam et al. (136) found that IF reduced infarct size, lowered mortality from focal ischemic stroke, and improved brain cell resistance to ischemic injury, involving upregulation of various neuroprotective proteins (neurotrophic factors, chaperones, antioxidants) and downregulation of pro-inflammatory factors (TNF, IL-6, and IL-1β). Fann et al. (77) showed that 4 months of daily 16 h fasting reduced the activation of NF-κB and MAPK signaling pathways in the infarcted tissue of mice after ischemia–reperfusion, decreased NLRP1 and NLRP3 inflammasome activity, and lowered IL-1β and IL-18 levels, reducing inflammation and tissue damage. Manzanero et al. (137) used a method similar to Fann et al. and found that pre-modeling IF led to weight loss and reduced circulating leptin levels, increased neurogenesis in the hippocampus and subventricular zone, and after ischemic injury, the infarct volume in the IF group was less than half that of the AL group, preventing the ischemia-induced decrease in circulating leptin levels. Stroke-induced cell proliferation and neurogenesis decreased in the IF group, possibly related to reduced cell death. Varendi et al. (138) studied the neuroprotective effects of short-term pre-operative fasting on a rat model of focal stroke. The study found that pre-operative fasting for 3 days reduced infarct volume in rats with severe focal stroke, and the neuroprotection was associated with the regulation of innate immunity: elevated circulating neutrophil chemokine ligand 1 before ischemia and reduced pro-inflammatory markers (TNF-*α* and its receptor, as well as downstream intercellular adhesion molecule-1) in the striatum after reperfusion. Jeong et al. (92) found that 2 weeks of IF reduced infarct volume and brain edema, improved neurological deficits, and reduced neuronal loss and apoptosis in MCAO rats. The mechanisms involved included reduced autophagy flux impairment and inhibition of apoptosis. Kim et al. (139) described transcriptional changes in the brain of IF mice and found that the brain transcriptome of AL group mice exhibited strong, sustained upregulation of deleterious genetic pathways under ischemic stroke, but the activation of these pathways was suppressed in the IF 16 h group. Hu et al. (140) found that post-operative IF reduced oxidative stress in the hippocampus of 2VO rats, as evidenced by decreased MDA concentration and reduced mRNA and protein levels of the reactive oxygen-generating enzyme NADPH oxidase 1. IF treatment also maintained GSH levels and SOD activity, as well as the levels of their upstream regulatory enzymes, thereby preserving antioxidant capacity. Additionally, post-operative IF prevented microglial activation and the elevation of sphingosine-1-phosphate receptor 1 and inflammatory cytokines in the hippocampus of 2VO rats. Poh et al. (141) used 14 to 16-week-old male C57BL/6NTac mice to study the effects of 4 months of IF (16 h of daily fasting) on inflammasome-mediated cell death in the cerebellum after chronic cerebral hypoperfusion (CCH). The study showed that IF reduced inflammasome activation and initiated pathways of apoptosis and pyroptosis in cell death. Song et al. (142) subjected rats to either AL or EODF for 3 months, followed by MCAO surgery, and found that IF or caloric restriction mimetics improved ischemic brain injury and microglial activation and enhanced *in vivo* angiogenesis. Caloric restriction mimetics or SIRT6 overexpression alleviated ischemic and reperfusion-induced brain injury *in vitro*. SIRT6 deacetylated H3K9ac and H3K56ac in HAPI cells and BMVECs to inhibit TXNIP. Downregulation of SIRT6 reversed the protective effect of caloric restriction mimetics during *in vitro* brain ischemia/reperfusion. Liu et al. (143) found that long-term IF improved neurogenesis after brain ischemia by promoting angiogenesis through activation of growth differentiation factor 11 (GDF11) signaling, thereby improving neurological function. Additionally, long-term IF stimulated endothelial cell proliferation, promoted local cerebral blood flow, and upregulated total vascular surface area and microvascular branch points through the GDF11/ALK5 pathway. Jeong et al. (144) subjected mice to daily 16 h fasting, with fasting periods occurring either during the dark phase (active-phase intermittent fasting) or the light phase (inactive-phase intermittent fasting). After 6 weeks of the dietary regimen, the mice underwent transient focal cerebral ischemia, and the study found that compared to the AL group, the active-phase intermittent fasting group exhibited better post-stroke motor and cognitive recovery and less infarction. Additionally, protection of dendritic spine density/morphology and increased expression of postsynaptic density protein 95 were observed in active-phase intermittent fasting. Chelluboina et al. (145) studied adult C57BL/6 male mice and found that compared to the AL group, the IF group showed improved motor function recovery, promoted a beneficial gut microbiota phenotype after transient MCAO, and had higher levels of SCFAs in fecal samples. Mersha et al. (146) found that EODF initiated 1 day after modeling promoted motor recovery in mice.

##### 5.1.3.3 Traumatic brain injury

TBI is characterized by structural or functional brain damage caused by external forces, and ferroptosis, a form of programmed cell death, is closely associated with TBI. Studies have shown that IF can effectively reduce lipid peroxidation and mitochondrial dysfunction, thereby alleviating cognitive impairments caused by injury. Yang et al. (49) conducted a study on 8- to 10-week-old C57BL/6 N mice, revealing that IF significantly improved cognitive function in TBI mice and reduced ferroptosis-related cellular damage. Specifically, IF markedly increased the expression of glutathione peroxidase 4 (Gpx4) and heat shock protein 1 (Hspb1), while inhibiting the elevation of ferroptosis-promoting factors such as Nfe2l2, Slc7a11, and Alox8. Additionally, Cao et al. (147) found that a one-month IF regimen, initiated 3 days post-TBI, enhanced the proliferation of hippocampal subgranular zone neural stem cells, increased neuropeptide Y (NPY) expression in the hippocampus, and improved cognitive performance in the Morris water maze test. These results indicate that IF mitigates TBI-induced cognitive deficits by promoting hippocampal neurogenesis through increased NPY expression. These findings provide new insights into the potential of IF as an intervention strategy for TBI.

##### 5.1.3.4 Epilepsy

Bruce-Keller et al. (110) divided 4-week-old adult SD male rats into AL and EODF groups and selected rats at four time points: 2, 4, 8, and 12 weeks after dietary intervention for bilateral dorsal hippocampal injection of kainic acid. The results showed that EODF significantly reduced hippocampal CA3 region neuronal loss, and Morris water maze tests (latency, exploration trials) showed significant differences between the EODF and AL groups, suggesting that 2–3 months of EODF could significantly reduce kainic acid-induced hippocampal damage and visuospatial memory impairment. Duan et al. (148) found that EODF significantly increased brain-derived neurotrophic factor (BDNF) levels in the hippocampus, cerebral cortex, and striatum of mice and reduced kainic acid-induced hippocampal neuronal damage. Contestabile et al. (102) found that 2-month-old male Wistar rats subjected to 6 months of EODF provided sufficient protection to GABAergic neurons in the hippocampus and olfactory-entorhinal cortex from kainic acid-induced degeneration. Sharma et al. (149) subjected 3-month-old male Wistar rats to 3 months of EODF and found that, compared to the AL group, EODF significantly reduced kainic acid-induced neuronal death in the hippocampal CA3 region and regulated the levels of different antioxidants and antioxidant enzymes in different brain regions, increased heat shock protein 70 (HSP70) expression, and counteracted kainic acid excitotoxicity. Youssef et al. (150) evaluated the effects of short-term (7–10 weeks) EODF on adult (6-month-old) SD rats and found that short-term EODF after adulthood had no effect on normal neuronal function (long-term potentiation) but significantly reduced kainic acid excitotoxicity. Hartman et al. (151) tested the anti-epileptic effects of dietary restriction regimens using epilepsy trials and found that the effects of IF varied with the epilepsy trial: IF increased epilepsy activity in the 6 Hz test and the maximal electroshock seizure test, provided no protective effect against pentylenetetrazol-induced epilepsy, but provided protection against kainic acid-induced epilepsy. Their study suggested that the potential anti-epileptic mechanisms of IF and the ketogenic diet might differ. Karimzadeh et al. (152) evaluated the protective effects of EODF on pentylenetetrazol-induced epilepsy rats through behavioral evaluation and histopathological examination and found that EODF reduced the density of black neurons in the hippocampal CA1 and CA3 regions and reduced the number of TUNEL-positive neurons in the hippocampus. These findings support the anti-epileptic and neuroprotective effects of EODF and suggest that the protective effects of EODF are strongest when intervention is initiated before excitotoxic damage occurs. Landgrave-Gómez et al. (153) found that TRF had anti-convulsive effects by prolonging the latency to forelimb clonic seizures, reducing the seizure severity score, and reducing the number of animals reaching status epilepticus. Additionally, TRF induced changes in signaling pathways regulating energy metabolism, including increased AMPK phosphorylation and decreased Akt kinase phosphorylation. TRF also significantly increased the concentration of *β*-hydroxybutyrate (β-HB), an endogenous inhibitor of histone deacetylases (HDACs). HDAC activity was significantly reduced, and histone 3 (H3) acetylation was increased in hippocampal homogenates from the TRF group. Dolce et al. (154) fed male NIH Swiss mice (3–4 weeks old) four different dietary regimens for 12–13 days and found that ketogenic diet (KD) mice were protected in the 6 Hz seizure test but exhibited more severe seizure scores in the kainic acid test (cohorts I and II), whereas IF mice showed the opposite. The study suggested that KD and IF do not share the same anti-epileptic mechanisms. Karimzadeh et al. (152) found that PF had anti-convulsive and neuroprotective effects in epileptic rats but emphasized that different PF regimens might have different effects. PF showed the strongest anti-convulsive effect when initiated before excitotoxic damage occurred. Armstrong et al. (155) studied the anti-convulsive effects of TRF in the 6 Hz mouse model. The study found that continuous 8 h TRF and 8hTRF plus weekend *ad libitum* feeding regimens temporarily increased seizure thresholds in the 6 Hz model after about 2 weeks, consistent with stable blood glucose levels during feeding and fasting periods.

### 5.2 Clinical studies

#### 5.2.1 Neurodegenerative diseases

Preliminary clinical studies suggest that IF may regulate cognitive function through various metabolic pathways, including ketone body synthesis and degradation, butyrate metabolism, pyruvate metabolism, and glycolysis and gluconeogenesis pathways (156). A 36-month prospective cohort study conducted by Ooi et al. (157) found that IF might improve cognitive function in elderly participants with mild cognitive impairment (MCI) through mechanisms such as antioxidant function, DNA damage, inflammation, and a limited set of metabolic biomarkers (insulin and HDL cholesterol). Kamel et al. (158) observed 24 PD patients during Ramadan fasting in 2016 and found no reports of severe side effects during fasting. Compared to before Ramadan, there were no significant changes in quality of life (PDQ 39), non-motor symptom scales, or severity indices after the fasting period. Phillips et al. (159) conducted a case report involving a 41-year-old male with progressive, worsening HD who followed a time-restricted ketogenic diet for 48 weeks. His motor symptoms, activities of daily living, comprehensive unified HD rating scale (cUHDRS) scores, most major HD-related behavioral problems, and quality of life improved.

#### 5.2.2 Autoimmune diseases

In a clinical trial involving MS patients, intermittent energy restriction altered blood lipid factors and gut microbiota, similar to the protective changes observed in mice (124). A preliminary randomized controlled study by Fitzgerald et al. (160) involving 36 MS patients found that a CR diet was a safe/feasible method for weight loss in MS patients and might be associated with improved mood health. Subsequent plasma sample non-targeted metabolomics studies found that larger changes in lysophosphatidyl and lysophosphatidylcholine metabolites in intermittent CR were associated with greater reductions in memory T cell subsets and greater increases in naive T cell subsets (161). Roman et al. (162) evaluated the safety and feasibility of several fasting-mimicking diets in MS patients and found that strict adherence to TRF dietary changes might be more feasible than calorie restriction. Damiani et al. (163) conducted a real-world multicenter study on 108 patients with moderate to severe plaque psoriasis who fasted during Ramadan and found that PASI scores significantly decreased after Ramadan fasting. Adawi et al. (164) studied the effects of Ramadan fasting on 37 patients with psoriatic arthritis and found that fasting was a predictor of decreased disease activity scores (DAS, BASDAI, LEI, and DSSI). The study also found that IL-17 therapy was an independent predictor of reduced LEI scores after fasting. Almutairi et al. (165) conducted a prospective observational study and found that Ramadan intermittent fasting had beneficial effects on disease severity in 121 patients with psoriasis, with statistically significant differences in fasting blood glucose, HDL, and triglycerides, and decreased PASI scores, with no serious health hazards. Ben et al. (166) evaluated the effects of Ramadan fasting on 36 patients with rheumatoid arthritis (RA) and 20 patients with spondyloarthritis (SpA) and found that IF had beneficial effects on RA activity but had a lesser impact on SpA patients, although overall improvements were observed. Further studies on RA patients (167) found that IF could rapidly improve RA activity, and the positive effects of this fasting regimen could last up to 3 months, suggesting that the recommended fasting interval could be estimated at 3 months. Goharifar et al. (168) evaluated the effects of Ramadan fasting on disease activity, health-related quality of life, and lipid profile in 40 patients with quiescent systemic lupus erythematosus (SLE). After 24.1 ± 5.4 (mean ± SD) days of fasting, anti-double-stranded DNA (anti-dsDNA) levels increased by 0.34 ± 0.41 mmol/dL in the case group compared to 0.07 ± 0.31 mmol/dL in the control group (*p* = 0.026). Similarly, C3 levels increased more significantly in the case group (16.8 ± 17.5 vs. 2.3 ± 13.2 mg/dL, *p* = 0.006). After 3 months of fasting, anti-dsDNA levels in the case group increased by 0.28 ± 0.46 mmol/dL, while in the control group, they decreased by 0.02 ± 0.43 mmol/dL (*p* = 0.04). Conversely, C3 levels returned to baseline levels. These changes were not accompanied by significant changes in disease activity and health-related quality of life. Ramadan fasting had no effect on lipid profile, except that total cholesterol levels decreased more slowly in the case group than in the control group (decreased by 16.4 ± 29.4 vs. decreased by 4.6 ± 23.9 mg/dL, *p* = 0.018).

#### 5.2.3 Neurotraumatic diseases

A randomized controlled study by Zheng et al. (169) found that an 8-week intermittent fasting intervention led to significant reductions in fasting blood glucose and weight in SCI patients but did not affect BMI, suggesting that IF could be considered a safe method. Guelpa et al. (170) first reported the use of IF to treat epilepsy, and in this case series, 15 patients did not follow the diet correctly, but 2 patients showed improvement before stopping the diet, and 4 patients showed some improvement. Hartman (171) conducted an IF intervention in 6 children aged 2–7 years with epilepsy who were poorly controlled by the ketogenic diet, and the study found that despite hunger-related adverse effects, 3 children persisted with the IF combined ketogenic diet treatment for 2 months, and 4 children experienced transient improvements in seizure control. Alqadi et al. (172) studied 37 adult patients with active epilepsy and found that fasting during Ramadan might have a positive impact on seizure control, with this effect persisting for up to 1 month after fasting, without affecting quality of life scores. A prospective observational study involving 321 patients with different seizure types and active epilepsy also found similar results (173).

## 6 Discussion

This review has summarized the multiple mechanisms by which IF affects fatty acid metabolic reprogramming and the neuroimmune microenvironment, and explored its potential applications in neurodegenerative, autoimmune, and neurotraumatic diseases. Current research findings indicate that IF significantly influences fatty acid metabolism, ketogenesis, immune regulation, and neuroprotective processes by activating multiple metabolic and cellular signaling pathways, thus presenting extensive health benefits and potential clinical applications.

Firstly, the role of IF in fatty acid metabolism is reflected in its dual regulation of fatty acid synthesis and breakdown. IF enhances metabolic flexibility and energy efficiency by promoting fatty acid oxidation and inhibiting its synthesis through the activation of AMPK and SIRT1 signaling pathways (9–13, 23, 24). The regulation of fatty acid metabolism is a key step in maintaining energy homeostasis and cellular stability, which is crucial for preventing and treating diseases related to metabolic disorders, such as obesity, diabetes, and cardiovascular diseases (8, 73).

Secondly, the impact of IF on the neuroimmune microenvironment is particularly noteworthy. Metabolites derived from fatty acid metabolism (such as ketone bodies and short-chain fatty acids) play significant roles in regulating neuroinflammation and immune responses (14–22). Ketone bodies not only serve as alternative energy sources but also activate the Nrf2 pathway to reduce oxidative stress, thereby protecting neurons from damage (29, 33–35). Moreover, the regulation of T-cell and microglial activity by short-chain fatty acids further strengthens the neuroprotective effects of IF on the neuroimmune system (19–22). This mechanism of regulating neuroinflammation through metabolic and immune interactions provides a theoretical basis for the potential intervention of neurodegenerative diseases, such as Alzheimer’s and Parkinson’s disease (48, 119).

The neuroprotective role of IF is also reflected in its effects on mitochondrial function. IF enhances mitochondrial biogenesis and reduces oxidative stress through the activation of AMPK and SIRT1 pathways, thereby protecting neurons from oxidative damage (23, 24, 50). Mitochondrial function regulation is crucial for maintaining cellular energy supply and reducing cellular stress, contributing to the significant neuroprotective potential of IF in the treatment of neurodegenerative and neurotraumatic diseases (86–88).

Furthermore, autophagy and apoptosis, as key processes in cellular homeostasis, are effectively modulated by IF. By enhancing autophagic activity and inhibiting unnecessary apoptosis, IF provides protective mechanisms in neurodegenerative conditions (33, 90–95). IF promotes neuronal survival and functional recovery by activating autophagy to clear damaged organelles, inhibiting apoptotic factors, and upregulating protective factors. These mechanisms suggest that IF could be an effective strategy for preventing and delaying the progression of neurodegenerative diseases (28, 49, 89).

Despite supportive evidence from animal studies and preliminary clinical trials regarding the application of IF in neurodegenerative, autoimmune, and neurotraumatic diseases, there are still many areas requiring further investigation. For instance, the long-term effects of IF on different populations (such as the elderly, individuals with metabolic disorders, and patients with neurological diseases) remain unclear. Moreover, the specific mechanisms of IF, especially the complex interactions of signaling pathways and their differential effects on various tissues and cell types, need to be further elucidated through randomized controlled trials. Future research should focus on unveiling the long-term safety and efficacy of IF, as well as exploring optimal protocols and personalized therapeutic potential for its clinical application.

Overall, IF demonstrates extensive health benefits and potential therapeutic applications through fatty acid metabolic reprogramming, neuroimmune regulation, mitochondrial optimization, and autophagy modulation. These mechanisms provide a solid foundation for the application of IF in the prevention and treatment of neurodegenerative diseases, autoimmune disorders, and neurotraumatic conditions. With more in-depth and comprehensive research in the future, IF holds promise as an effective, low-cost clinical intervention that can help improve metabolic health and neurological function.
